# Augmentation Cystoplasty in a Girl With Early B-cell Factor 3 (EBF3)-Related Syndrome

**DOI:** 10.7759/cureus.63997

**Published:** 2024-07-06

**Authors:** Yusuke Kiyama, Fumi Matsumoto, Satoko Matsuyama, Futoshi Matsui

**Affiliations:** 1 Urology, Osaka Women's and Children's Hospital, Osaka, JPN

**Keywords:** children, augmentation cystoplasty, bladder dysfunction, hadds, ebf3-related syndrome

## Abstract

Early B-cell factor 3 (EBF3)-related syndrome, also known as hypotonia, ataxia, and delayed development syndrome (HADDS), is a recently recognized neurodevelopmental disorder frequently associated with bladder dysfunction. Despite bladder dysfunction possibly caused by detrusor sphincter dyssynergia, previous studies reported relatively accepted bladder compliance in patients with HADDS. We present the first case of bladder augmentation, in the English literature, in a girl with EBF3-related syndrome due to poor bladder compliance with clean intermittent catheterization and anticholinergic medication.

## Introduction

Early B-cell factor 3 (EBF3)-related syndrome was first recognized in 2017 [1-3], and to date, approximately 100 cases have been reported [4-8], exhibiting decreased muscle tone, motor dysfunction, and neurodevelopmental delay. It is also known as hypotonia, ataxia, and delayed development syndrome (HADDS) and exhibits not only characteristic facial features but is commonly also associated with bladder dysfunction possibly caused by detrusor sphincter dyssynergia (DSD) [6-10]. Although urinary diversion by vesicostomy was applied in cases with severe bladder dysfunction diagnosed in infancy, no cases of augmentation cystoplasty have been reported. Here, we present a case of bladder dysfunction in a girl with EBF3-related syndrome that resulted in successful urinary control with clean intermittent catheterization (CIC) after ileocystoplasty.

## Case presentation

**Figure 1:**
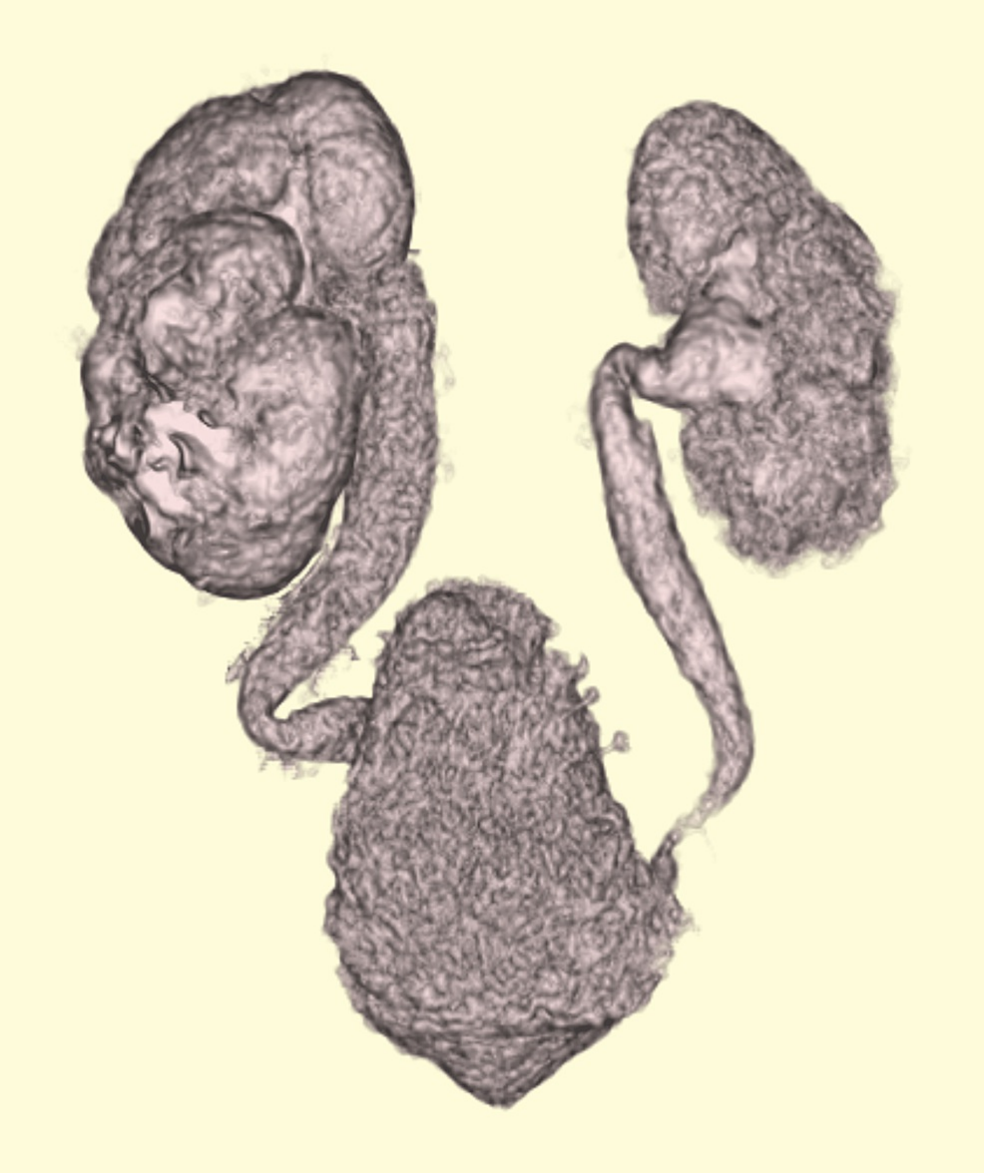
Computed tomography 3D reconstructed computed tomography showed bilateral tortuous hydroureters and bladder deformity.

A 10-month-old girl was referred to our hospital for the evaluation of recurrent febrile urinary tract infections (fUTI) and bilateral upper urinary tract dilation with bladder deformity. The patient's prenatal and family histories were unremarkable. She was delivered vaginally at 37 weeks of gestation. Her birth weight was 3620 g. Although esotropia became evident, no other anomalies, including neurological or anorectal malformations, were detected. She had a history of fUTI at four and eight months of age. Imaging studies at a local hospital revealed bilateral hydronephrosis associated with tortuous hydroureters and bladder deformity (Figure 1).

No vesicoureteral reflux (VUR) was detected on voiding cystourethrography.

**Figure 2:**
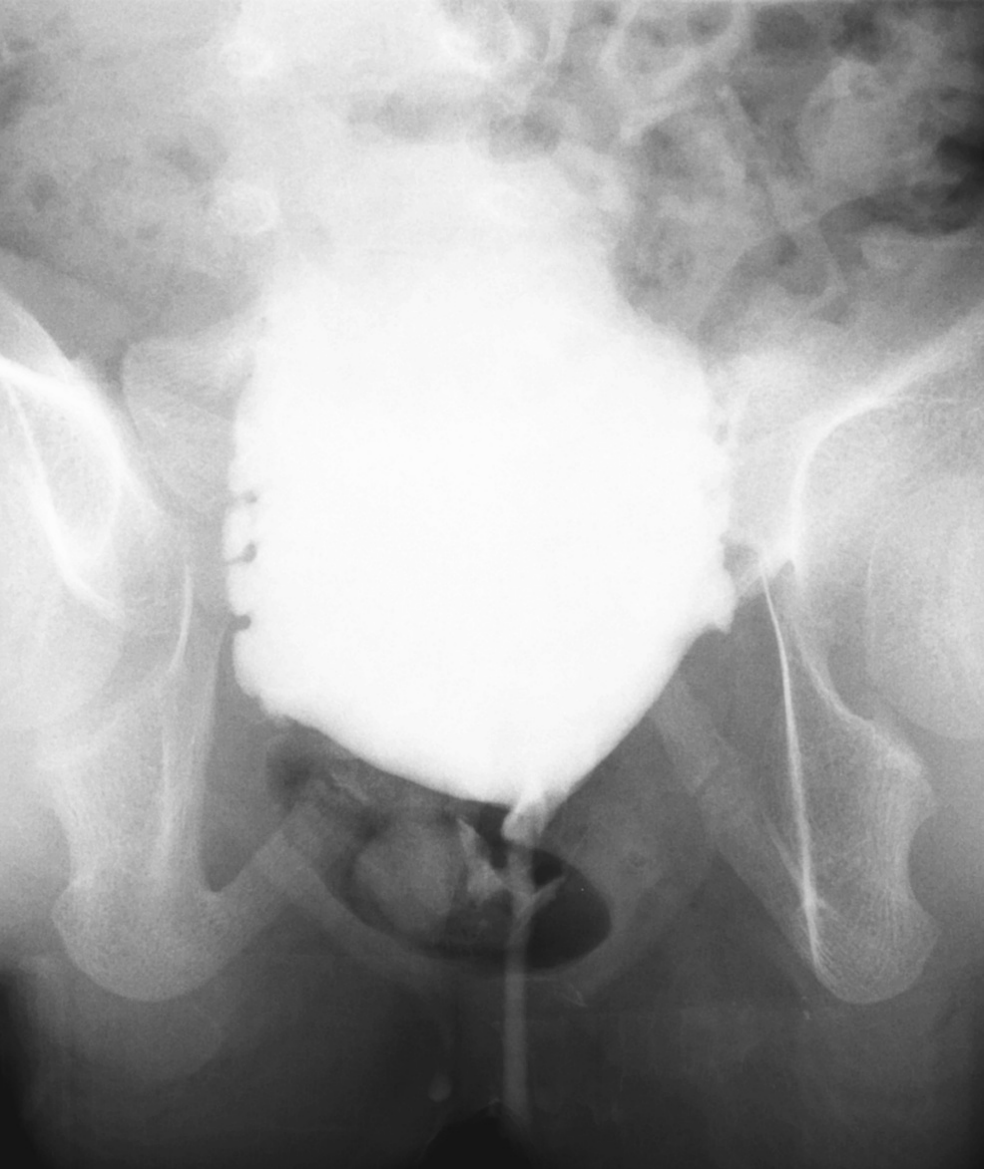
Cystography in a voiding phase No vesicoureteral reflux was detected.

At the time of referral, she was well-nourished despite mild constipation. Physical examination revealed no abnormal findings in the external genitalia or back. She had lower abdominal distension that resolved after bladder emptying. Urethral catheterization was performed without any difficulty. Brain computed tomography and spinal magnetic resonance imaging revealed no abnormalities. To treat severe bladder dysfunction of unknown etiology, we initiated CIC combined with anticholinergic medication, continuous antibiotic prophylaxis, and bowel treatment. Although urinary diversion by cutaneous vesicostomy was recommended due to non-improvement of upper urinary tract dilation and recurrence of fUTI, her family did not accept it because of concerns regarding body image. Bladder function deteriorated over time. Serial video-urodynamic studies showed decreased bladder capacity and poor bladder compliance with detrusor overactivity and the absence of VUR (Figure 2).

At the age of six years and four months, bladder capacity and end-fill detrusor pressure were 96 ml and 36 cm H2O, respectively.

**Figure 3:**
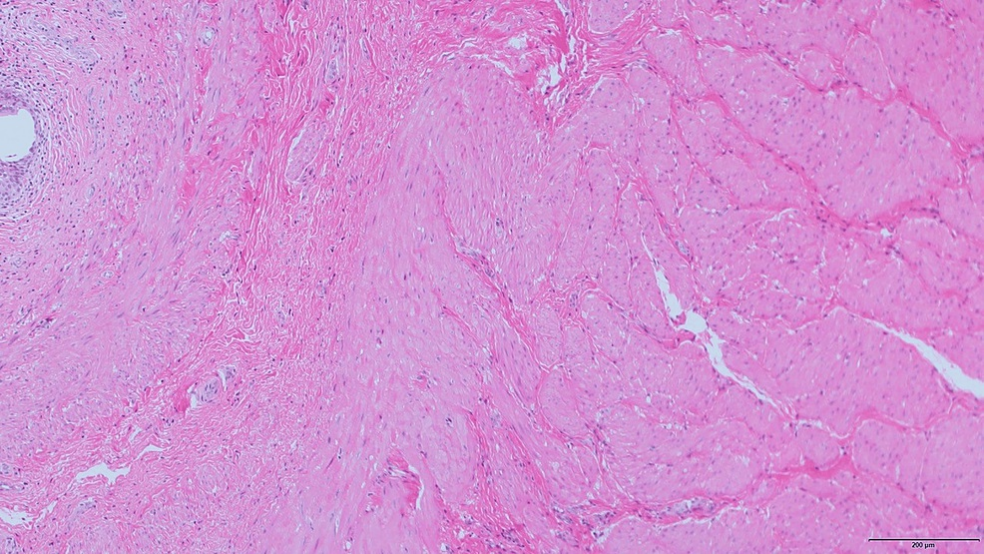
Histopathologic section of the bladder Hematoxylin and eosin stain demonstrated severe trabeculation of the bladder only.

Augmentation cystoplasty using the ileal segment was performed at the age of six years and eight months. Intraoperative findings of the bladder wall were not noteworthy except for trabeculation, which was confirmed by pathological examination (Figure 3).

The postoperative course was uneventful. She was dry on clean intermittent self-catheterization, without upper urinary tract dilation or fUTI recurrence at four years follow-up.

Because of clinical features including gait instability and mild psychomotor developmental delay since the age of three years and six months, whole-exome sequencing was performed using genomic DNA extracted from the proband and parents at the age of six years and eight months. A de novo variant (c.411+1G>A), for which loss-of-function is a known mechanism of the disease, was identified in EBF3, leading to the diagnosis of EBF3-related syndrome.

## Discussion

EBF3, located on chromosome 10q26, encodes a transcription factor involved in neuronal differentiation and maturation. Heterozygous pathogenic variants and single-gene deletions of EBF3 cause a neurodevelopmental disorder known as HADDS [1-5]. A recent meta-analysis showed both sexes are equally affected, and 90% of the diagnoses were made by 19 years of age [6]. Although some autosomal dominant HADDS cases were reported, 66% of individuals exhibited a de novo inheritance pattern of pathogenic EBF3 variants [6].

The clinical manifestations of HADDS are broad and include congenital hypotonia, delayed psychomotor development, intellectual disability, and dysmorphic craniofacial features. In addition, urogenital abnormalities such as bladder dysfunction associated with upper urinary tract dilation with or without VUR, UTI, and cryptorchidism are common. Based on their six patients with HADDS and a literature review of 46 patients, Batie et al. [7] reported that two-thirds of the patients had urological manifestations. Notably, the rate of bladder dysfunction was higher in their cohort (four of six patients, 67%) than in the literature cohort (nine of 46 patients, 19.7%). They reported that, owing to the rarity and recent discovery of this syndrome, urological manifestations may be underdiagnosed and underreported. Nishi et al. also reported a high incidence of UTI and urinary tract abnormalities in infants with HADDS and indicated that a neurogenic bladder associated with delays in motor/skills is a clue for the early diagnosis of HADDS [9]. Based on urodynamic findings, Batie et al. [7] demonstrated a high prevalence of DSD in patients with HADDS. While EBF3 is important in neurodevelopment, it also affects muscle development in vertebrates [11]. Because EBF3 is widely expressed throughout the body, they hypothesized that some EBF3 variants affect the relaxation of the external urethral sphincter, leading to DSD, which may cause bladder dysfunction, secondary VUR, and UTI. Despite bladder dysfunction possibly caused by DSD, bladder compliance in patients with HADDS was relatively acceptable in their cohort and literature review. To date, no patients who underwent bladder augmentation have been reported. However, as they mentioned, all children with severe bladder dysfunction diagnosed during infancy were managed with vesicostomy. In the present case, progressive deterioration of bladder compliance was observed with CIC and anticholinergic medications. Although early urinary diversion contributes to the prevention of secondary damage from DSD, poor bladder compliance may be the primary cause of the effect of EBF3 on the detrusor muscle in patients with HADDS.

Ileocystoplasty was safely performed in our patient. The intraoperative and histopathological findings were similar to those of other neurogenic bladders. Augmentation cystoplasty provides an adequate urinary reservoir for patients with HADDS when necessary.

## Conclusions

We present the first case of ileocystoplasty in a girl with EBF3-related syndrome. Augmentation cystoplasty may be considered as an alternative surgery for urinary diversion in some cases of HADDS with exhibiting poor bladder compliance resistant to conservative therapy.
